# A145 UNDERWATER EMR FOR LARGE FLAT DYSPLASTIC COLORECTAL LESIONS IN PATIENTS WITH CHRONIC ULCERATIVE COLITIS

**DOI:** 10.1093/jcag/gwad061.145

**Published:** 2024-02-14

**Authors:** R Yu, R Barclay

**Affiliations:** Internal Medicine, The University of British Columbia Faculty of Medicine, Vancouver, BC, Canada; Internal Medicine, The University of British Columbia Faculty of Medicine, Vancouver, BC, Canada

## Abstract

**Background:**

Patients with chronic ulcerative colitis are at increased risk of colorectal cancer, in part due to increased prevalence of dysplastic lesions. To reduce CRC risk, endoscopic resection is preferable to surgery if feasible. However, large flat lesions can be challenging to remove endoscopically. We report our experience with underwater EMR for large, flat ulcerative colitis-associated dysplastic lesions.

**Aims:**

To evaluate the safety and efficacy of UEMR for treatment of flat/sessile colorectal lesions with dysplasia, without overt signs of invasive cancer. To analyze rates of complications and outcomes for patients undergoing UEMR and compare to alternative methods of treatment for similar lesions.

**Methods:**

We reviewed our IBD patient database from 2021 to 2023 for ulcerative colitis patients referred for large (≥ 20 mm diameter) flat/sessile colorectal lesions without overt signs of invasive cancer, who subsequently underwent underwater EMR. The procedure was performed by an endoscopist with extensive experience in UEMR for large colorectal lesions.

**Results:**

Eleven *de novo* lesions in 8 patients (4 female; age 58 ± 14) were successfully treated with single-session UEMR. Mean lesion size was 42 ± 22 mm (range 20 to 80 mm); all morphologically flat (Paris IIa or IIb). Three cases were resected *en bloc*; the remainder piecemeal. Pathology showed tubulovillous adenoma, N=4 (36%); tubular adenoma, N=5 (45%); traditional serrated adenoma, N=1 (9%); sessile serrated lesion, N=1, (9%); high-grade dysplasia, N=1 (9%). There were no cases of invasive cancer. Endoscopic follow-up available for 5 patients over 10.4 ± 2 months showed benign diminutive recurrent/residual lesions in 2 cases, both amenable to UEMR. There were no procedural complications.

**Conclusions:**

In this series of large, flat dysplastic CUC-associated lesions, UEMR was safe and effective, obviating surgery and endoscopic submucosal dissection.

**Funding Agencies:**

None

